# Supplementary material for: Biosecurity and Hygiene Procedures in Pig Farms: Effects of a Tailor-Made Approach as Monitored by Environmental Samples
Source: Animals (Basel). 2023 Apr 5;13(7):1262. doi: 10.3390/ani13071262 (PMC10093544; doi:10.3390/ani13071262)
Supplement: Supplementary file 1 [file animals-13-01262-s001.zip › Supplementary material.pdf]

animals

Article

Biosecurity and hygiene procedures in pig farms: effects of a tailor-made approach as monitored by environmental samples

Supplementary material

Annalisa Scollo [1,*], Alice Perrucci[1], Maria Cristina Stella[1], Paolo Ferrari[2], Patrizia Robino[1], Patrizia Nebbia[1]

[1] Department of Veterinary Sciences, University of Torino, 10095 Grugliasco, Torino, Italy
[2] CRPA Research Centre for Animal Production, 42121, Reggio Emilia, Italy
* Correspondence: annalisa.scollo@unito.it

Table S1. General description of the 10 farms involved in the bacteriological investigation. Production phase: breeding/nursery = from the farrowing site to approximately 30 kg of body weight; fattening = from approximately 30 kg of body weight to slaughter-170 kg of body weight. Size: in case of breeding/nursery = number of productive sows and/or number of weaned piglets present the day of the visit; in case of fattening sites = number of farmed pigs present the day of the visit. The third visit was made 12 months after the first.

			Biosecurity scores at the first visit (improvement at the third visit)					
**Farms**	**Production phase**	**Size**	**Total biosecurity, %**	**Public zone, %**	**Public/professional transition, %**	**Professional zone, %**	**Professional/herd transition, %**	**Herd zone, %**
1	Fattening	3500	53.3 (+1.7)	60.4 (0.0)	46.9 (+1.0)	60.0 (+7.5)	31.6 (0.0)	67.6 (0.0)
2	Breeding/nursery	1800	56.8 (+4.2)	60.4 (0.0)	46.9 (+3.1)	60.0 (+7.5)	43.3 (+10.6)	73.1 (0.0)
3	Breeding/nursery	1300	64.8 (+2.5)	85.4 (0.0)	75.0 (+3.1)	60.0 (+5.0)	36.8 (+2.7)	66.7 (+1.8)
4	Fattening	5200	70.9 (+6.7)	87.5 (0.0)	80.2 (+4.2)	70.0 (+22.5)	40.8 (+3.9)	75.9 (+2.8)
5	Fattening	2800	60.2 (+1.3)	50.0 (0.0)	70.8 (+4.2)	57.5 (+2.5)	48.7 (0.0)	74.1 (0.0)
6	Fattening	800	49.2 (-2.3)	72.9 (-10.4)	36.4 (-3.1)	35.0 (+5.0)	36.8 (0.0)	64.8 (-2.8)
7	Fattening	1300	49.9 (+1.1)	52.1 (+6.2)	44.8 (0.0)	55.0 (0.0)	32.9 (0.0)	64.8 (-0.9)
8	Fattening	1600	54.2 (0.0)	54.2 (0.0)	58.3 (0.0)	55.0 (0.0)	27.6 (0.0)	75.9 (0.0)
9	Breeding/nursery	1800	58.3 (-0.5)	56.2 (0.0)	62.5 (0.0)	42.5 (-2.5)	52.6 (0.0)	77.8 (0.0)
10	Breeding/nursery	1200	79.7 (+4.2)	87.5 (0.0)	86.4 (+4.2)	82.5 (+10.0)	51.3 (+5.3)	90.7 (+1.9)

Table S2. Livestock-associated Methicillin-resistant staphylococci (LA-MRS) and extended spectrum β-lactamase (ESBL) producing *E. coli* in environmental samples collected at the beginning of the study (first visit) in 10 swine farms. Isolations from environmental samples collected at the end of the study (third visit, 12 months later) are also reported for six farms.

			**First visit n Positive Samples/n Tested (%)**		**Third visit n Positive Samples/n Tested (%)**	
**Farms**	**Productive phase**	**Isolation**	**Before cleaning**	**After cleaning**	**Before cleaning**	**After cleaning**
1	Fattening	LA-MRS	4/4 (100.0)	2/4 (50.0)	-	-
		ESBL-*E. coli*	1/4 (25.0)	0/4 (0.0)	-	-
2	Breeding/ nursery	LA-MRS	3/4 (75.0)	1/4 (25.0)	0/4 (0.0)	0/4 (0.0)
		ESBL-*E. coli*	3/4 (75.0)	0/4 (0.0)	0/4 (0.0)	0/4 (0.0)
3	Breeding/ nursery	LA-MRS	3/4 (75.0)	4/4 (100.0)	1/4 (25.0)	0/4 (0.0)
		ESBL-*E. coli*	2/4 (50.0)	0/4 (0.0)	1/4 (25.0)	0/4 (0.0)
4	Fattening	LA-MRS	4/4 (100.0)	4/4 (100.0)	-	-
		ESBL-*E. coli*	3/4 (75.0)	0/4 (0.0)	-	-
5	Fattening	LA-MRS	4/4 (100.0)	1/4 (25.0)	4/4 (100.0)	4/4 (100.0)
		ESBL-*E. coli*	2/4 (50.0)	0/4 (0.0)	1/4 (25.0)	0/4 (0.0)
6	Fattening	LA-MRS	0/4 (0.0)	0/4 (0.0)	-	-
		ESBL-*E. coli*	0/4 (0.0)	0/4 (0.0)	-	-
7	Fattening	LA-MRS	0/4 (0.0)	2/4 (50.0)	4/4 (100.0)	2/4 (50.0)
		ESBL-*E. coli*	0/4 (0.0)	0/4 (0.0)	0/4 (0.0)	0/4 (0.0)
8	Fattening	LA-MRS	3/4 (75.0)	4/4 (100.0)	-	-
		ESBL-*E. coli*	0/4 (0.0)	1/4 (25.0)	-	-
9	Breeding/ nursery	LA-MRS	4/4 (100.0)	4/4 (100.0)	4/4 (100.0)	3/4 (75.0)
		ESBL-*E. coli*	0/4 (0.0)	0/4 (0.0)	1/4 (25.0)	0/4 (0.0)
10	Breeding/ nursery	LA-MRS	2/4 (50.0)	4/4 (100.0)	4/4 (100.0)	4/4 (100.0)
		ESBL-*E. coli*	1/4 (25.0)	0/4 (0.0)	2/4 (50.0)	2/4 (50.0)
**Total**		LA-MRS	27/40 (67.5)	26/40 (65.0)	17/24 (70.8)	13/24 (54.2)
		ESBL-*E. coli*	12/40 (30.0)	1/40 (2.5)	5/24 (20.8)	2/24 (8.3)

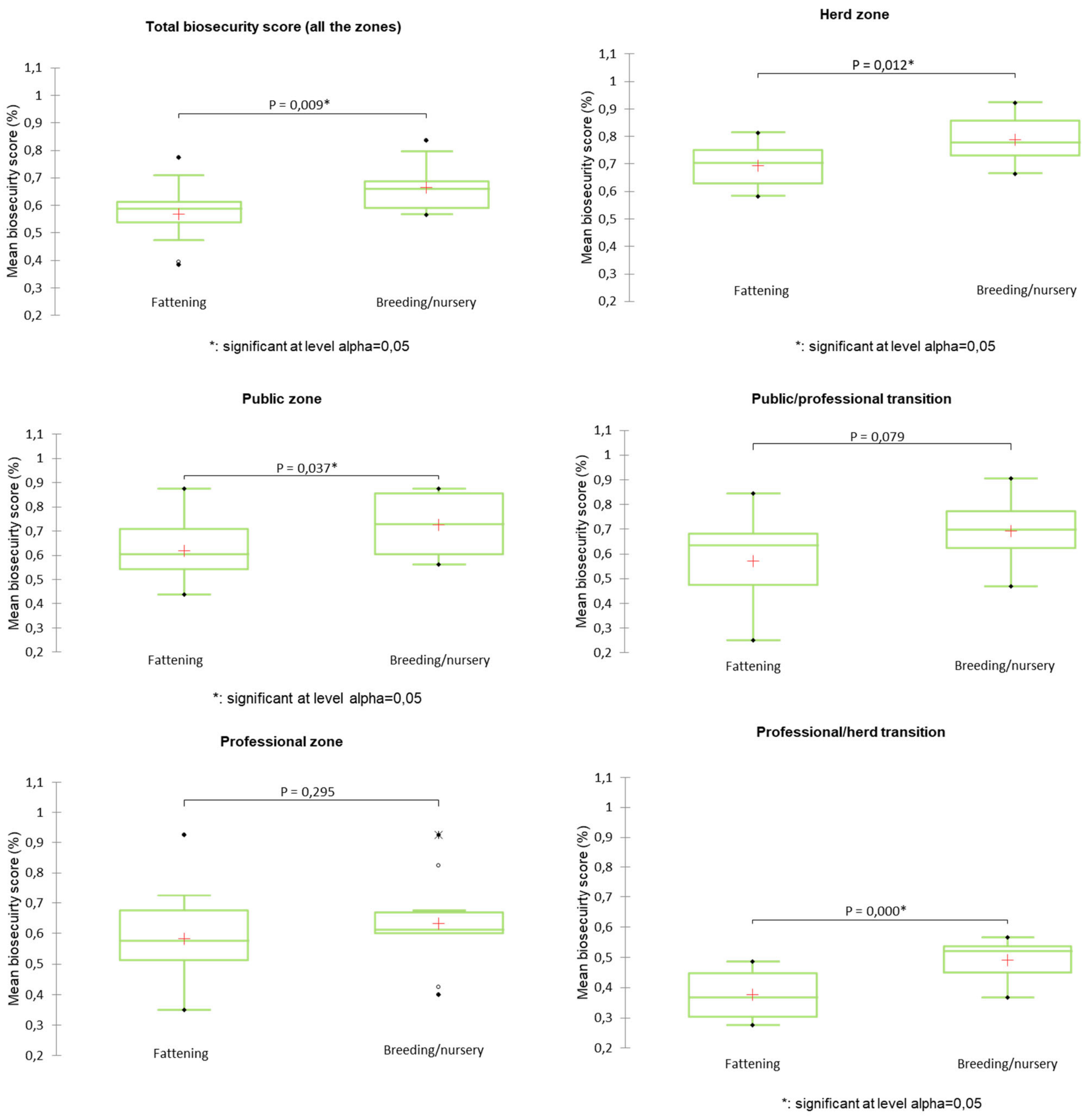

Figure S1. Boxplots of the distribution of biosecurity scores for farm zones in breeding/nursery (n = 5) and fattening sites (n = 15). In the boxes, the thick horizontal line represents the median biosecurity score, whereas the base and the top of the boxes are the first and third quartiles, respectively. Whiskers extend to a maximum of 1.5 times the interquartile range. Points represent extreme values.
